# Copy number changes and methylation patterns in an isodicentric and a ring chromosome of 15q11-q13: report of two cases and review of literature

**DOI:** 10.1186/s13039-015-0198-4

**Published:** 2015-12-21

**Authors:** Qin Wang, Weiqing Wu, Zhiyong Xu, Fuwei Luo, Qinghua Zhou, Peining Li, Jiansheng Xie

**Affiliations:** Shenzhen Maternity and Child Healthcare Hospital, 3012 Fuqiang Road, Shenzhen, Guangdong China; Department of Genetics, Yale School of Medicine, New Haven, CT USA; First Affiliated Hospital, Biomedical Translational Research Institute, Jinan University, Guangzhou, Guangdong China

**Keywords:** Isodicentric chromosome, Ring chromosome, 15q11-q13, Array comparative genomic hybridization (aCGH), Methylation-specific multiplex ligation-dependent probe amplification (MS-MLPA)

## Abstract

**Background:**

The low copy repeats (LCRs) in chromosome 15q11-q13 have been recognized as breakpoints (BP) for not only intrachromosomal deletions and duplications but also small supernumerary marker chromosomes 15, sSMC(15)s, in the forms of isodicentric chromosome or small ring chromosome. Further characterization of copy number changes and methylation patterns in these sSMC(15)s could lead to better understanding of their phenotypic consequences.

**Methods:**

Routine G-band karyotyping, fluorescence in situ hybridization (FISH), array comparative genomic hybridization (aCGH) analysis and methylation-specific multiplex ligation-dependent probe amplification (MS-MLPA) assay were performed on two Chinese patients with a sSMC(15).

**Results:**

Patient 1 showed an isodicentric 15, idic(15)(q13), containing symmetrically two copies of a 7.7 Mb segment of the 15q11-q13 region by a BP3::BP3 fusion. Patient 2 showed a ring chromosome 15, r(15)(q13), with alternative one-copy and two-copy segments spanning a 12.3 Mb region. The defined methylation pattern indicated that the idic(15)(q13) and the r(15)(q13) were maternally derived.

**Conclusions:**

Results from these two cases and other reported cases from literature indicated that combined karyotyping, aCGH and MS-MLPA analyses are effective to define the copy number changes and methylation patterns for sSMC(15)s in a clinical setting. The characterized genomic structure and epigenetic pattern of sSMC(15)s could lead to further gene expression profiling for better phenotype correlation.

## Background

The low copy repeats (LCRs) clustered in the chromosome 15q11-q13 region are known breakpoints 1 to 5 (BP1-5) for meiotic non-allelic homologous recombination which results in interstitial deletions and duplications [1]. Deletions of this region account for approximately 70 % of patients with Prader-Willi syndrome (PWS, OMIM#176270) and Angelman syndrome (AS, OMIM#105830). Reciprocal duplications of 15q11-q13 can cause autism, developmental delays, intellectual disability, ataxia, seizures, and behavioral problems (OMIM#608636). The PWS/AS critical region (PWACR) of 15q11-q13 contains many imprinting genes and shows the parental-origin effects [2]. In addition to intrachromosomal rearrangements, small supernumerary marker chromosomes 15, sSMC(15)s, in the forms of an inverted duplication (inv dup) or an isodicentric chromosome (idic) and a small ring chromosome, were also derived from rearrangements of the LCRs of 15q11-q13 [3]. The phenotypic consequences of these sSMC(15) are associated with their genomic structure, parental-origin imprinting effects and level of mosaicism [4].

Most sSMC(15)s take the form of a dicentric inv dup and can be classified into two groups: small sSMC(15)s and large sSMC(15)s. The small sSMC(15)s have breakpoints at the BP1 or BP2 proximal to the critical region and usually clinically irrelevant, while the large sSMC(15)s frequently extend beyond the BP3 to include the critical region and are frequently associated with abnormal phenotypes [5–17]. However, unexpected level of structural complexity including asymmetrical breakpoints, unequal size of inverted arms, and multiple types of atypical rearrangements among sSMC(15)s were noted [9, 12, 14, 15]. Previous studies showed that *de novo* sSMC(15)s characterized molecularly were of maternal origin [5, 7, 9, 10, 17]. It has been recognized that maternal duplication of this region will produce abnormal phenotype but paternal duplication carriers are commonly unaffected. However, recent studies showed that patients with paternal duplication of 15q11-q13 may also have mild abnormal phenotype [8, 17]. In addition to the genomic structure and parental origin, the level of mosaicism might also alter the risk associated with an abnormal phenotype. A mitigate effect correlating the mild phenotype of motor and speech development delay with the percentage and the type of cell lineages containing the sSMC(15) was suggested [10, 13, 15]. However, results from a large case series showed that about 60 % percent mosaic sSMC cases with clinical abnormalities had no direct correlation to the level of mosaicism in the peripheral blood and there is no simple relationship between clinical abnormalities and sSMC mosaicism [4].

The application of array comparative genomic hybridization (aCGH) analysis has proven very effective in defining the breakpoints, copy number changes, and gene content for sSMC(15)s [11, 12, 14–17]. Recently, methylation-specific multiplex ligation-dependent probe amplification (MS-MLPA), a rapid and cost-effective technique with high specificity and sensitivity, has been introduced for genetic analysis of copy number changes and methylation patterns [18–21]. In this study, we present copy number changes and methylation pattern from an isodicentric chromosome 15 and a small ring chromosome 15. Review of literature found five reports with combined copy number and methylation analyses on 34 cases of sSMC(15)s and two cases of small ring chromosome 15 [17, 22–25]. These results demonstrate that combined karyotype, FISH, aCGH and MS-MPLA analyses could be used in a clinical setting effectively to define genomic structure, parental origin and level of mosaicism for sSMC(15)s.

## Results

Patient 1 is a 3-year-old girl. She was born at 41 weeks of gestation from an uneventful pregnancy and delivered by Caesarean section. Her birth weight was 3,550 g (75th percentile) and birth length was 51 cm (85th percentile). She showed head control at 6 months, standing with aid at 18 months, and walking not steadily at 26 months. Her verbal language was nearly absent and no visual contact. The daily life was completely taken care by the family. She showed no dysmorphic features and no record of seizures but was hypotonia and impulsive. She failed to follow instructions and lacked response to commands. Electroencephalography (EEG) study and nuclear magnetic resonance imaging (MRI) were normal. The parents were healthy and non-consanguineous. The father was 40-year-old and the mother was 42-year-old at the time of her birth. Parental chromosome studies were normal.

**Fig. 1:**
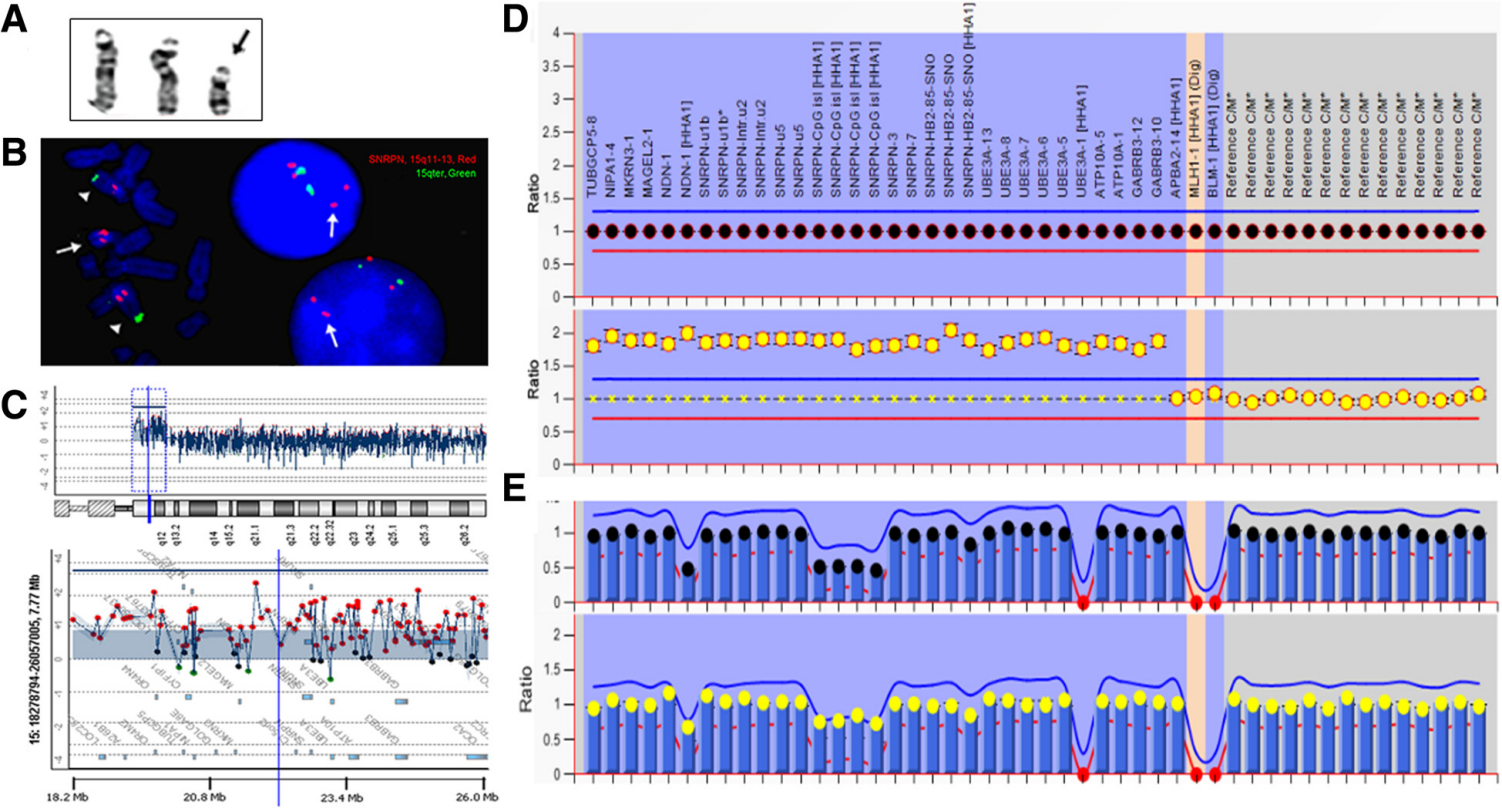
Karyotyping, FISH, aCGH and MS-MLPA results in patient 1. **a**. The chromosome image shows a normal pair of chromosome 15 and the extra idic(15). **b**. Metaphase and interphase FISH results show two copies of the *SNRPN* gene in the idic(15) (*SNRPN* red, 15qter green). **c**. The aCGH chromosome view (up) and gene view (bottom) reveal the breakpoint location and a 7.7 Mb duplication. **d**. The MS–MLPA pattern shows a peak height ratio value of 2 (four copies) in chromosome 15 (bottom) in comparison with a ratio value of 1 (two copies) from a normal control (upper). **e**. The MS-MLPA pattern indicates a methylation percentage of 0.75 in four *SNRPN* recognition sites in patient 1 (bottom) in comparison of 0.5 from a normal control (upper)

For patient 1, karyotyping analysis showed a supernumerary isodicentric chromosome 15, 47,XX,+idic(15)(pter → q13.1::q13.1 → pter), in all cells examined (Fig. 1a). FISH test was performed using dual color probes for the SNRPN gene at 15q11.2 and a control locus at 15qter. Of the 20 metaphase cells analyzed, the normal chromosomes 15 showed positive hybridization signals on the targeted loci from both probes and the idic(15) had two strong signals from the SNRPN probe but no signal from the control probe. Of the 50 interphases examined, four signals for the SNRPN probe and two signals for the control probe were noted (Fig. 1b). The result confirmed that the idic(15) contained two copies of the *SNRPN* gene region. The aCGH result indicated a 7.7 Mb duplication of chromosome 15q11-q13 (chr15:18,362,355-26,110,139) including genes from *A26B1* to *HERC2*. The log_2_ ratio (L2R) was 0.885, indicating that the idic(15)(q13.1) was composed of two copies of the 15q11-q13 region with a breakage-fusion event occurred at BP3 (Fig. 1c). The MLPA result showed four copies for this chromosomal fragment by an increased mean peak height ratio of 2.0 (Fig. 1d). For the MS-MLPA in a normal control, the four probes for the *SNRPN* gene (a maternally methylated sequence containing a *HhaI* restriction site) decreased half of the peak height ratio, indicating the presence of one *Hha I* digested paternal unmethylated copy and another *Hha I* undigested maternal methylated copy. In patient 1, the MS-MLPA result showed a one-fourth decrease of the peak height ratio after *Hha I* digestion, indicating the presence of one copy unmethylated paternal *SNRPN* and three copies of methylated maternal *SNRPN* (Fig. 1e). These results indicated that the idic(15) was symmetric and of maternal origin.

Patient 2 was a six-year-old girl. She was born at 39 weeks of gestation from an uneventful pregnancy and delivered by Caesarean section. She could sit without aid at age one year but walk clumsy and stumbled at 25 months. Her language ability was limited. She attended special educational training but made no much progress. She had intellectual disability, autistic like behaviors, hyperphagia and hyperactivity but no dysmorphic features. Sleep problem and epileptic seizure were not known in patient history. According to her parents, the girl could follow simple instructions and fetch small things. She could eat almost by herself but never achieved sphincter control. Her parents were healthy and they were 23-year-old at the time of her birth.

**Fig. 2:**
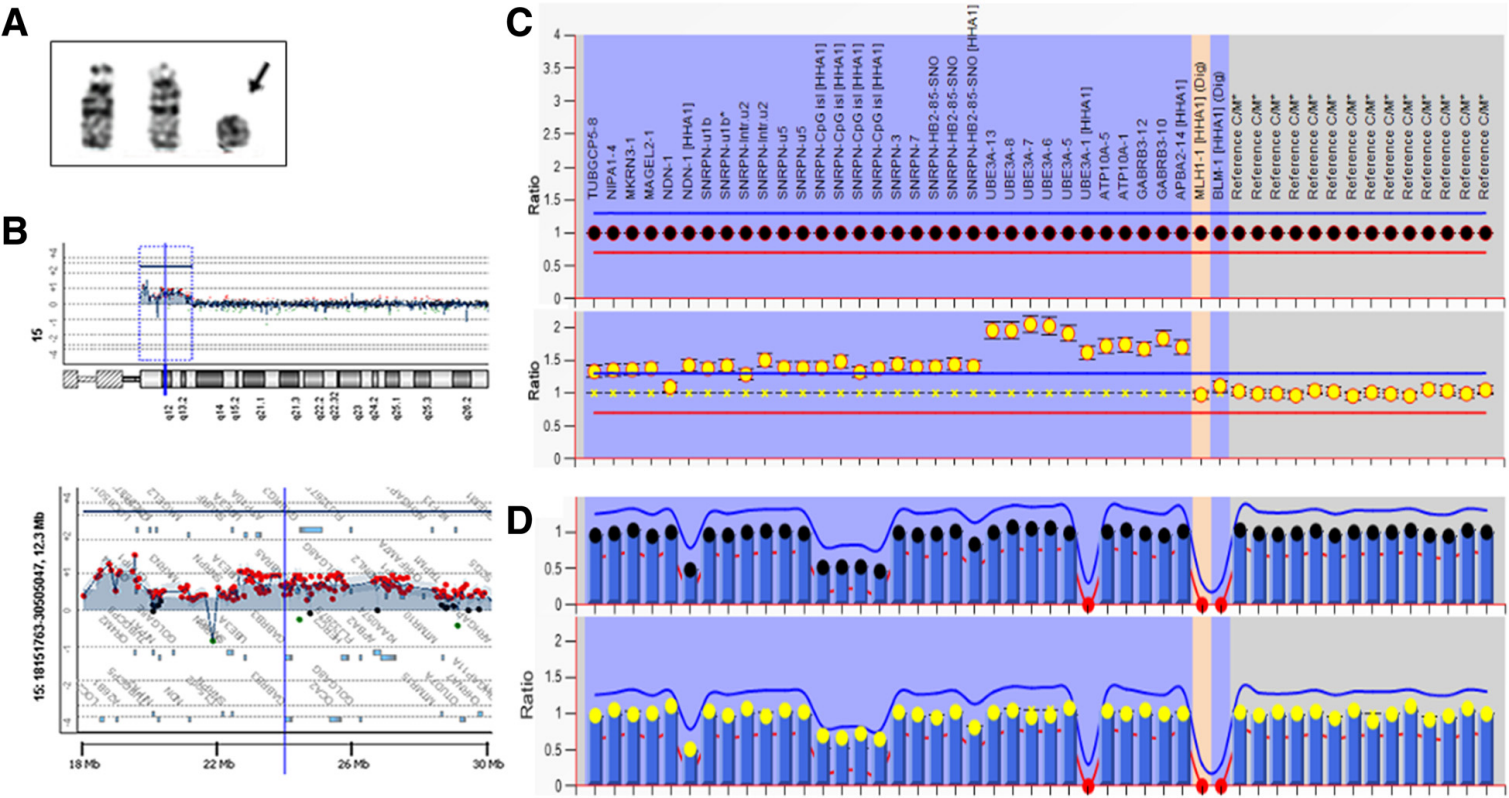
Karyotyping, aCGH and MS-MLPA results in patient 2. **a**. The chromosome image shows a normal pair of chromosome 15 and the extra r(15). **b**. The aCGH chromosome view (up) and gene view (bottom) reveal the breakpoint location and a 12.3 Mb. **c**. The MS-MLPA pattern shows peak height ratio value of 1.5 to 2.0 (three or four copies) in chromosome 15 (bottom) in comparison with a ratio value of 1 (two copies) from a normal control (upper). **d**. The MS-MLPA pattern indicates methylation aberration of 0.75 in four *SNRPN* recognition sites in patient 2 (bottom) in comparison with 0.5 from a normal control (upper)

For patient 2, chromosome analysis performed on 100 metaphase cells from cultured peripheral blood lymphocytes showed a mosaic pattern for a supernumerary small ring chromosome 15, 47,XX,+r(15)(q13)[32]/46,XX[68] (Fig. 2a). The aCGH analysis revealed unique copy number changes in the 12.3 Mb region of 15q11-q13 (chr15:18,362,355-30,701,573) encompassing genes from *A26B1* to *CHRNA7*. Starting from the proximal to the distal end at BP5, a 1.571 Mb tetrasomic segment of 15q11.1-q11.2 (chr15:18,362,355-19,934,192, L2R:1.000, proximal to BP1 with polymorphic copy number variants), a 2.404 Mb trisomic segment at 15q11.2 (chr15:20,418,129-22,821,963, L2R:0.360, from BP1 to between BP2/BP3), a 4.974 Mb tetrasomic segment of 15q11.2-q13.1 (chr15:23,020,445-27,994,906, L2R:0.727, from BP2/BP3 to BP3/BP4), followed by a 1.791 Mb trisomic segment (chr15: 28,910,278-30,701,573, L2R:0.350, from BP4 to BP5) were delineated (Fig. 2b). The MLPA result revealed an increased mean peak height ratio of 1.5 in segment from gene *TUBGCP5* to *SNRPN* and a ratio of 2.0 in segment from *UBE3A* to *APBA2*. The results indicated that the r(15) had alternative two-copy and one-copy segments (Fig. 2c). The MS-MLPA result showed a one-fourth decrease of the peak height ratio after digestion, indicating that the duplication segment within the r(15) was methylated and of maternal origin (Fig. 2d).

## Discussion and conclusion

**Fig. 3:**
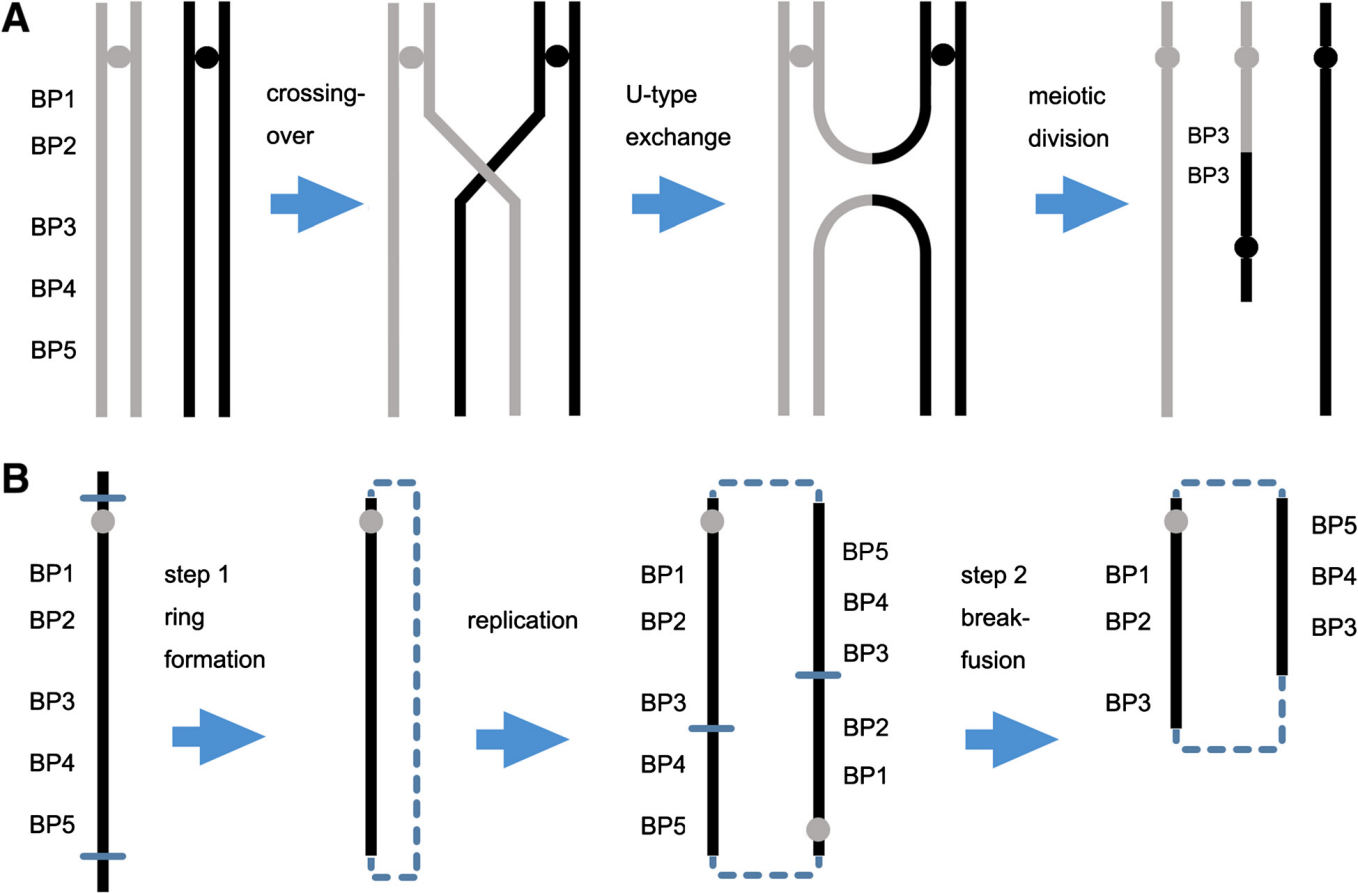
Mechanisms for the idic(15) and ring 15. **a**. A schematic drawing shows the U-type exchange during meiosis for the formation of the idic(15) with a BP3::BP3 fusion. **b**. A schematic drawing shows the two-step process for the formation of r(15) from the initial ring formation with break-fusion at BP5, the formation of double ring through replication, and subsequent asymmetric breakage-fusion for segmental duplication and deletion (thin line for breakpoint, dash line for joining point)

Currently, more than 1300 similar sSMC(15) cases (published or not) are collected in the online sSMC database (http://ssmc-tl.com/sSMC.html). Carefully checking the website and review of literatures found five reports with combined karyotype, aCGH/SNP and methylation analyses on 34 cases of idic(15) and two cases of small r(15) [17, 22–25]. The genomic structures and methylation patterns from these cases and our two cases are summarized in Table 1. For the formation of *de novo* idic(15), different types of breakage-fusion events including symmetrical BP3::BP3 and BP4::BP4 and asymmetric BP3::BP4, BP3::BP5 and BP4::BP5 were noted (Table 1). These observations indicated that the *de novo* idic(15)s originated from maternal meiotic crossing-over event between paired or mis-paired LCRS of homologous chromosomes in pachytene and followed by non-disjunction in the subsequent divisions [3]. Several modes of formation for inv dup or idic chromosome have been proposed. The most plausible mode of formation is the U-type exchange resulting from crossover mistakes of chromatids of two homologous chromosomes during meiosis [3] (Fig. 3a). Supernumerary small ring chromosome for the 15q11-q13 is an uncommon chromosomal abnormality and also likely derived from breakage and fusion event at the LCRs of 15q11-q13. However, the small r(15) from the two cases in the literature and our patient 2 showed break-fusions occurred between BPs (BP2/BP3 or BP3/BP4). The complex copy number changes and the variable breakage-fusion points within the r(15) may be explained by a two-step process including initial ring formation by a break-fusion event at the LCRs, an intermediate double ring from ring DNA replication, and a secondary asymmetric break-fusion event to introduce segmental duplications and deletions between BPs (Fig. 3b) [26, 27]. Therefore, for small supernumerary r(15), ring structure instability and secondary rearrangements should be considered.

**Table 1:** A summary of sSMC(15) defined by karyotype, aCGH or SNP, and methylation analyses

SMC15	Test Methods				Patient Number	Age	Gender	Inheritance	BP Fusion	Methylation	References
	G-banding	aCGH/SNP	FISH	Methylation							
trc(15), idic(15)	+	Nimblegen	+	MS-SB	2	11y, 26y	M, F	de novo	BP3::BP3, BP4::BP5	Maternal	[22] Hogart A, et al., 2009
rea(15), inv dup(15)	+	Affymetrix	+	MS-PCR	2	5y, 9y	M, M	de novo	BP4::BP5, BP4::BP5	Maternal	[23] Yang J, et al. 2013
inv dup(15) or idic(15)	+	Agilent & Illumina	+	MS-MLPA	8	1.7y-14.5y	M(3), F(5)	de novo	BP3::BP3,BP2-BP3::	Maternal	[17] Ageeli EA, et al. 2014
der(15)t(15q;6p)					1	7y	M	paternal carrier	BP2-BP3::	Paternal	
del(15)[14]/psu dic(15)[4]	+	Agilent	+	MS-PCR	1	2y	F	de novo	BP3::BP3	Maternal	[24] Tan E-S, et al. 2014
idic(15)	+	Agilent	+	MS-MLPA	20	3 m-23y	M(13), F(7)	1 de novo, 19 unk	BP3::BP3 or BP4, BP4::BP4 or BP5	Maternal	[25] Aypar U, et al. 2014
idic(15)	+	Agilent	+	MS-MLPA	1	3y	F	de novo	BP3::BP3	Maternal	This report
r(15)	+	Agilent	+	MS-MLPA	2	1d, 7y	M, M	unk	BP2/BP3::,BP3::BP3	Maternal	[25] Aypar U, et al. 2014
r(15)	+	Agilent	-	MS-MLPA	1	6y	F	de novo	BP5::BP5> > BP2/BP3::BP3/BP4	Maternal	This report

*Abbreviations*: *MS* methylation sensitive, *SB* Southern blot; + = yes; − = not; m = month; y = year; unk, unknown

Our two cases and almost all reported *de novo* cases of idic(15)s showed a genomic structure including PWACR and a methylation pattern of maternal origin [5–17, 22–25]. As reported from previous analyses, clinical phenotypes for sSMC(15)s are related with the duplication region containing the PWACR and the maternally derived homologue of chromosome 15q [13, 17]. A comparison of clinical features between our patients and among those previously reported cases with similar size of duplication noted that patient 2 showed a relatively mild phenotype despite a larger size containing genes from BP1 to BP5. The presence of normal cells from the mosaic ring sSMC(15) might alleviate the severity of the clinical manifestation. Since routine karyotyping analysis was only done for cells from peripheral blood culture, the percentage of the mosaic r(15) in other tissues was not known. Micro-invasive methods to access other types of tissues, especially muscular and neurologic tissues, are needed to evaluate the mosaic pattern for sSMC(15)s. The gene content within sSMC(15)s and the parental-origin imprinting effects could be the determine factors affecting the phenotype [22, 28]. Patient 1 had a 7.7 Mb BP1-BP3 duplication which contains genes involving in developmental or neurological diseases. The BP1-BP2 region contains *NIPA1, NIPA2* and *CYFIP1* genes which are associated with the central nervous system development or function [29–31]. The BP2-BP3 region contains paternally expressed genes *MKRN3, MAGEL2, NDN* and *SNRPN;* these four genes are implicated in the autism disorder [17, 32]. The maternally expressed *UBE3A* gene is exclusively-expressed in brain tissue and the neurodevelopmental complexities are associated with increased *UBE3A* in dup15q syndrome [33, 34]. The *NDN* gene is an imprinted gene expressed exclusively from the paternal allele, which is associated with neurological and muscular disorder and implicated as a negative growth regulator in human cancer [35, 36]. Patient 2 had a 12.3 Mb duplication involving BP1-BP5 region that extending to gene *CHRNA7*. The clinical phenotype like speech delay, hyperphagia, hyperactivity, mental retardation, no facial dysmorphism and no epilepsy may be influenced by gain of dosage of *CHRNA7* [17, 37, 38]. In addition to the gene dosage effect, the gene expression may also contribute to the variability of the phenotypes which were influenced in unexpected ways through epigenetic changes [22]. Further elucidation of cellular functions and molecular pathways of the genes within the BP1-BP5 duplication region will facilitate better phenotype prediction and therapeutic intervention.

Several molecular methods including Southern blot analysis on methylation sensitive restriction sites, MS-PCR, sequencing of bisulfate-treated DNA, MS-PCR and MS-MLPA, have been introduced to define methylation pattern for sSMC(15)s [21, 22, 39]. Southern blot and sequencing methods are more time consuming and expensive. MS-PCR may show more variation in copy number quantitation. The present study and several reports have demonstrated that MS-MLPA is a robust, high-throughput, rapid and inexpensive approach with high specificity and sensitivity [22–25]. It provides an efficient way to simultaneously detect copy number changes and DNA methylation within 15q11-q13 in a semi-quantitative manner [39]. Taken together, combined cell-based karyotyping and FISH to detect the chromosome structure and mosaic pattern with DNA-based aCGH and MS-MLPA for copy number changes and methylation patterns should be recommended for clinical analysis of sSMC(15). Practice guidelines for PWS/AS and analytic algorithms for sSMC(15)s using this combined methods have been proposed [25, 39].

In conclusion, we have defined the copy number changes and methylation pattern in an idic(15) and a r(15) from two Chinese patients by karyotyping, aCGH, and MS-MLPA analysis. The results revealed that the idic(15) with a BP3::BP3 fusion and a r(15) likely resulting from secondary breakage-fusion between BP2/BP3 and BP3/BP4 were maternally derived. Variable spectrum of neurodevelopmental phenotype might be explained by the gene dosage and epigenetic imprinting effects from these sSMC(15)s.

## Methods

### Patients

Two patients were referred for genetic evaluation of developmental delay, speech retardation and intellectual disabilities at the genetic counseling clinic in Shenzhen Maternal and Child Healthcare Hospital. This study was approved by the hospital’s Institutional Review Board and written informed consents were obtained from their parents.

### Karyotype analysis

Chromosome analysis was performed on G-banded metaphases from cultured peripheral blood lymphocytes according to the laboratory’s standard protocols. An extended analysis of 100 G-banded metaphase cells was performed to allow the detection of equal or greater than 3 % of mosaicism with 95 % confidence interval [40].

### FISH analysis

Fluorescence in situ hybridization (FISH) analysis was performed on metaphase chromosomes and interphase nuclei using dual color probes for the *SNRPN* gene at 15q11.2 and a control locus at 15qter (Cytocell Inc.) following the manufacturer’s instruction. Hybridization signal patterns were analyzed on twenty metaphase cells and 50 interphase cells. FISH probe preparation, in situ hybridization, signal scoring, and image capture were performed as previously described [41].

### Array comparative genomic hybridization (aCGH)

Genomic DNA was extracted from the peripheral blood using the Gentra Puregene Blood kit (Qiangen, Valencia, CA, USA). DNA concentration was measured using a NanoDrop spectrophotometer (ND-1000, Thermo Fisher Scientific Inc., Waltham, Mass., USA), and DNA quality was verified by agarose gel electrophoresis. For each case, 2 ug of patient genomic DNA was used following the protocol from the SurePrint G3 Human CGH 8x60K Microarray Kit (Agilent Technologies, Santa Clara, CA, USA). DNA labeling, sex-matched test/control hybridization, post hybridization washes, image scanning, and data analysis were processed as previously described [39]. The base pair positions for detected genomic imbalances were designated according to the March 2006 Assembly (NCBI36/hg18) in the UCSC Human Genome browser (http://genome.ucsc.edu/).

### MS-MLPA

MLPA reagents were obtained from MRC-Holland (Amsterdam, The Netherlands; SALSA MLPA kit ME028). The ME028 Kit can be used to detect copy number changes and to analyze the CpG island methylation of the 15q11 region in a semi-quantitative manner. The Kit contains 32 probes specific for sequences in the PWACR and 14 reference probes outside the region. Four of the PWACR specific probes in the *SNRPN* gene contain a recognition site for the methylation sensitive *HhaI* enzyme and can be used for the presence of aberrant methylation patterns in the 15q11 locus. The *NDN* gene also contains methylation probes while it has a known tendency to over-digest resulted in variable results. The experiment procedures were performed following the manufacturer’s protocol [18, 42]. The MS-MLPA data was imported into the software Coffalyser.Net (designed by MRC-Holland) to analyze both the copy number variation and the methylation profile.
